# Functions of Gut Microbiota Metabolites, Current Status and Future Perspectives

**DOI:** 10.14336/AD.2022.0104

**Published:** 2022-07-11

**Authors:** Juan Liu, Yuzhu Tan, Hao Cheng, Dandan Zhang, Wuwen Feng, Cheng Peng

**Affiliations:** ^1^State Key Laboratory of Southwestern Chinese Medicine Resources, School of Pharmacy, Chengdu University of Traditional Chinese Medicine, Chengdu 611137, China.; ^2^Key Laboratory of the Ministry of Education for Standardization of Chinese Medicine, Chengdu University of Traditional Chinese Medicine, Chengdu 611137, China.

**Keywords:** gut microbiota metabolites, intestinal barrier, energy metabolism, immune response, circadian rhythm, short-chain fatty acids

## Abstract

Gut microbiota, a collection of microorganisms that live within gastrointestinal tract, provides crucial signaling metabolites for the physiological of hosts. In healthy state, gut microbiota metabolites are helpful for maintaining the basic functions of hosts, whereas disturbed production of these metabolites can lead to numerous diseases such as metabolic diseases, cardiovascular diseases, gastrointestinal diseases, neurodegenerative diseases, and cancer. Although there are many reviews about the specific mechanisms of gut microbiota metabolites on specific diseases, there is no comprehensive summarization of the functions of these metabolites. In this Opinion, we discuss the knowledge of gut microbiota metabolites including the types of gut microbiota metabolites and their ways acting on targets. In addition, we summarize their physiological and pathologic functions in health and diseases, such as shaping the composition of gut microbiota and acting as nutrition. This paper can be helpful for understanding the roles of gut microbiota metabolites and thus provide guidance for developing suitable therapeutic strategies to combat microbial-driven diseases and improve health.

The gut microbiota is a community of microorganisms that dwell in a mutualistic relationship with hosts in gastrointestinal tract. Although the importance of gut microbiota has been recognized for a long time, it is only in recent decades that our understanding of gut microbiota began to surge because of the progresses in genomics, metabolomics, and other technologies such as culturomics [1]. Integrative analysis of gut microbiota of human subjects, antibiotic-treated, germ-free, or gnotobiotic animals have revealed that the gut microbiota plays a causal, or at least a correlative relationship in the development of diseases such as non-alcoholic fatty liver disease, obesity, inflammatory bowel disease, Alzheimer’s disease, Parkinson’s disease, allergy, and depression [2,3]. In addition to the direct roles in development of diseases, the gut microbiota can interact with the drugs that are taken orally and impact their efficacy and toxicity [4]. Because of the importance of gut microbiota in disease development and modification of drug efficacy, the gut microbiota has become an indispensable target that needs to be considered in medical area.

In normal condition, the gut microbiota is enclosed within the gastrointestinal lumen by intestinal barrier, a structure composed of epithelial cells, mucus, commensal bacteria, immune cells, and antibodies [5]. To circumvent this spatial limitation, the gut microbiota evolves a strategy of releasing different classes of metabolites to exert the effects on hosts and other gut bacteria. In this event, comprehensive identification of gut microbiota metabolites and investigation of their roles have naturally become important for researchers. With the help of advanced metabolomic tools such as ultra-performance liquid chromatography-tandem mass spectrometry, a large group of metabolites have been identified, such as short-chain fatty acids (SCFAs), bile acids, and choline metabolites [6,7]. These metabolites can induce a series of physiological and pathological functions on hosts and other bacteria, such as modulation of energy metabolism, nutrition absorption, and regulation of gut microbiota composition [8]. These microbiota-host and bacteria-bacteria metabolic dialogs are essential for understanding the roles of gut microbiota in maintenance of health and promotion of diseases.

Currently, there are many reviews about the specific functions and mechanisms of gut microbiota metabolites on specific diseases, such as the mechanisms of SCFAs on inflammatory bowel diseases and their functions on host metabolism [9-11]. However, there is no comprehensive summarization of the functions of gut microbiota metabolites, which has limited our understanding of the gut microbiota and our combat against microbial-associated diseases. In this Opinion, focusing on the functions of gut microbiota metabolites, we first give brief introduction on the classification and production of gut microbiota metabolites. Then, we emphasize the functions of gut microbiota-derived metabolites, especially the functions of regulating energy metabolism, local and systemic immune system, and neural activity. At last, the perspectives on future research directions are discussed.

## 2. Gut microbiota metabolites

### 2.1. Classification of gut microbiota metabolites

It is estimated that the gut microbiota contains about 10^15^ microbial cells and more than 22 million microbial genes, both of which exceed the cells and genes of human [12]. With these genes, the gut microbiota can synthesize a myriad of enzymes with versatile capabilities to ferment a variety of compounds that have escaped from the digestion of human enzymes or compounds that are indigestible by human enzymes such as fibers [13]. As a result, the gut microbiota can produce a battery of metabolites with wide spectrum of bioactivities. According to the origination, the gut microbiota metabolites can be broadly divided into three types: (1) metabolites that are produced by gut microbiota directly from diets, such as SCFAs and indole derivatives; (2) metabolites that are generated by the host and modified by gut microbiota, such as secondary bile acids; (3) metabolites that are produced *de novo*, such as polysaccharide A [14]. Many of the gut microbiota metabolites share similar chemical structure, and they showed similar functions on hosts. According to the chemical structures and their functions, we have listed the major groups of metabolites, the typical metabolites and their targets inTable 1. It should be noted that because the chemical structure of gut microbiota metabolites is diverse and thus the classification here is not very strict. In addition, a specific gut microbiota metabolite can act on multiple organs or tissues, and thus possesses multiple functions. For example, butyrate can act as energy resource and nutrition for colonocyte, modulate the intestinal barrier and systemic immune response [20]. Therefore, the functions of gut microbiota metabolites are not isolated, and should be treated systemically. Among all the gut microbiota metabolites, the most extensively studied metabolites are SCFAs, bile acids, and amino acid-derived metabolites.

**Table 1:** Typical gut microbiota metabolites and their roles in health and diseases.

Groups	Typical metabolites	Typical targets	Specific functions	Typical diseases associated	Ref.
**Short-chain fatty acids**	Acetate, propionate, butyrate, hexanoate, isovalerate, isobutyrate, 2-methylpropionate, valerate	Directly act on GPR41, GPR43, GPR109A, GPR81, GPR91, HDAC1 and HDAC3	Regulation of gut microbiota composition, gut barrier integrity, appetite, energy homeostasis, gut hormone production, circadian clocks; inhibit proinflammatory cytokines; stimulate water and sodium absorption; modulate systemic immune response	Diabetes, obesity, pancreatitis, nonalcoholic fatty liver disease, hypertension, atherosclerosis, chronic kidney disease, ulcerative colitis, radiation proctitis, Crohn’s disease, colorectal cancer, autism spectrum disorder, sclerosis, Parkinson’s disease, asthma, diarrhea	[15-21]
**Bile acids**	Cholate, hyocholate, deoxycholate, taurohyocholate, ursodeoxycholate, taurocholate, tauro- *α*-muricholate, glycocholate, hyodeoxycholate, tauro- *β*-muricholate, lithocholate, taurodeoxylcholate	Directly act on FXR, VDR, PXR/SXR, constitutive androstane receptor (CAR), TGR5, sphingosine 1-phosphate receptor 2 (S1PR2), formyl-peptide receptor (FPR), muscarinic acetylcholine receptor (mAChR)	Facilitate lipid and vitamin absorption; regulation of gut microbiota composition, gut hormones, intestinal immunity, intestinal electrolyte and fluid balance, gut motility, lipid homeostasis, glucose homeostasis, amino acid homeostasis, circadian clocks; influence neurotransmission and physiology	Primary biliary cholangitis, primary sclerosing cholangitis, obesity, nonalcoholic fatty liver disease, non-alcoholic steatohepatitis, atherosclerosis, ulcerative colitis, cancer, hepatic encephalopathy, multiple sclerosis, Alzheimer's disease, Parkinson's disease, traumatic brain injury, stroke and amyotrophic lateral sclerosis	[8,22-25]
**Gases**	H_2_S, H_2_, CO_2_, CH_4_, NO	NO targets soluble guanylate cyclase, H_2_S cause conformational changes of target proteins by sulfhydration	CH_4_ slows gut motility; H_2_S regulates gut inflammation, motility, epithelial secretion and susceptibility to infections; NO mediates gastric mucosal protection and regulate mucosal blood flow	Parkinson’s disease, colitis, ulcer	[26-31]
**Tryptophan and indole derivatives**	Indole-3-lactic acid, indole acetic acid, indole-3-acetamide, indole pyruvic acid, indoxyl sulfuric acid, indole, serotonin	Directly targeting on AhR and PXR	Influence the gut microbial spore formation, drug resistance, biofilm formation, and virulence; regulate intestinal barrier functions, gut hormone secretion, gut motility, systemic immune response	Ulcerative colitis, Crohn’s disease, obesity, stroke, mucosal candidiasis, autism spectrum disorder, Alzheimer’s disease, Parkinson's disease, migraine, schizophrenia, irritable bowel syndrome	[32-36]
**Choline metabolites**	TMA, methylamine, dimethylglycine, dimethylamine,	Direct target unknown, but can activate NF-кB, protein kinase C (PKC), NLRP3 inflammasome	Inhibits bile acid synthesis; promote inflammation, thrombosis; affects myocardial hypertrophy and fibrosis; exacerbates mitochondrial dysfunction	Nonalcoholic fatty liver disease, obesity, atherosclerosis, diabetes, heart failure, hypertension	[37-39]
**Vitamins**	Vitamin B2, Vitamin B3, Vitamin B5, Vitamin B6, Vitamin B9, Vitamin B12, vitamin K	Vitamin receptors	Involved in cellular metabolism; modulate immune function and cell proliferation; supply vitamins for hosts	Vitamin associated diseases such as schizophrenia, autism, and dementia	[40,41]
**Neurotransmitters**	Dopamine, catecholamines, 5-HT, and GABA	Adrenergic receptors, 5-HT receptors, GABA receptors	Regulate gut motility, memory and stress responses, immune function of nervous system	Parkinson's disease, autism	[27,42,43]
**Lipids**	Conjugated fatty acids, cholesterol, phosphatidylcholines, triglycerides, LPS	LPS targets directly on TLR4	LPS triggers systemic inflammation; conjugated fatty acids regulate hyperinsulinemia, immune system, lipoprotein profiles; cholesterol acts as material bases for bile acid synthesis.	Diabetes, obesity, nonalcoholic fatty liver disease, hyperinsulinemia, hypercholesterolemia, chronic hepatitis C.	[7,44,45]
**Others**	Ethanol; triphosadenine; lantibiotic such as ruminococcin A and cytolysin; microcin such as microcin B17; organic acids such as benzoate and hippurate; polyamines such as cadaverine, and spermidine	Triphosadenine activate P2X and P2Y receptors	Enhance or damage gut barrier; regulate intestinal or systemic immune response; act as antibiotics to modulate gut microbiota composition; supply the nutrients; be toxic to host cells	Fatty liver disease, *C. difficile* and *H. pylori* infections, irritable bowel syndrome, ulcerative colitis	[7,46-48]

### 2.2. Typical gut microbiota metabolites in a nutshell

During the past decade, a wealth of literatures has paid attention to SCFAs and SCFAs have become the cynosure of all the gut microbiota metabolites. SCFAs are saturated aliphatic acids with number of carbons ranges from one to six. Acetate, propionate, and butyrate are the most common SCFAs produced by gut microbiota. Most SCFA are generated by fermenting of carbohydrates that have escaped the digestion of human enzymes in stomach and small intestine, and in a much lesser extent by fermenting undigested protein-derived branched chain amino acids (BCAAs) [49]. The undigested proteins and peptides that contain BCAAs can be metabolized typically to branched-chain fatty acids such as 2-methylbutyrate and iso-valerate [49]. The SCFAs are rapidly absorbed by colonocytes in large intestine via hydrogen-dependent or sodium-dependent monocarboxylate transporters following their production [50]. In colonocytes, a large part of SCFAs function as energy source, and the remaining SCFAs can be transported into the circulation system and other tissues such as brain, heart, and lung [51]. The targets of SCFAs include G protein-coupled receptor (GPR) 41, GPR43, GPR109A, GPR81, and GPR91 which are also named as free fatty acid receptor 3 (FFAR3), FFAR2, hydroxycarboxylic acid receptor (HCA) 2, HCA1, and succinate receptor 1, correspondingly [20]. SCFAs can also target nuclear class I histone deacetylases (HDACs) including HDAC1 and HDAC3, which are mainly correlated to the anti-inflammatory phenotype [52].

Bile acids are cholesterol derived amphipathic and water-soluble metabolites of saturated hydroxylated C-24 sterols that are originally synthesized by hepatocytes and transformed by gut microbiota [53]. In the liver, primary bile acids are produced from cholesterol by the classic or alternative pathway, the former of which plays major role [54]. In the classic pathway, 7 *α*-hydroxylase is the rate-limiting enzyme for synthesis of primary bile acids [55]. Chenodeoxycholic acid and cholic acid are the main hepatic bile acids synthesized in human and can conjugate to taurine or glycine to form bile salts [56]. Of note is that bile acids show obvious species-specific differences. For example, the primary bile acids generated by human are chenodeoxycholic acid and cholic acid, whereas rodents mainly produce cholic acid, chenodeoxycholic acid and muricholic acids [56]. Primary bile salts are synthesized in the liver, deposited in the gallbladder and discharged into the duodenum. Primary bile acids in intestinal tract can be reabsorbed by host and delivered back to liver, a process termed enterohepatic circulation, to maintain the bile acid pool [23]. In terminal ileum and colon, the primary bile acids that escape enterohepatic circulation can be transformed by gut microbiota into secondary bile acids mainly via bile salt hydrolases and 7 *α*-dehydroxylases [22,23]. The bile acids nuclear receptors include farnesoid X receptor (FXR), vitamin D3 receptor (VDR), pregnane X receptor/steroid and xenobiotic-sensing receptor (PXR/SXR), constitutive androstane receptor, and the membrane receptors include Takeda G-protein receptor 5 (TGR5), sphingosine 1-phosphate receptor 2, formyl-peptide receptor, and muscarinic acetylcholine receptor [8].

The proteins and peptides that escape digestion of hosts can undergo the metabolism of gut microbiota, and produce various bioactive compounds that includes, but not limited to, ammonia, amines, sulfides, nitrogen compounds, indoles, phenols, *p*-cresol sulfate, and precursors to branched-chain fatty acids [57]. Among all these compounds, tryptophan and tryptophan derivatives have caught the eyes of a large group of researchers. In the gastrointestinal tract, unabsorbed tryptophan can be transformed by the gut microbiota into tryptamine, skatole, indole and indole derivatives, such as indole-3-propionic acid, 3-methyl-indole, and indoxyl sulfate [36]. Many of these metabolites can act as ligands for aryl hydrocarbon receptor (AhR), a protein that can trigger a wide range of effects on the hosts such as modulation of metabolism, immunity, and social behavior [34,35]. Some amino acids can be sequentially metabolized by gut microbiota and hosts, as epitomized by carnitine, an amino acid that is abundant in red meat. Carnitine can be metabolized to trimethylamine (TMA) in the gut lumen by gut microbiota, and then be converted into trimethylamine-N-oxide (TMAO) by flavin-containing monooxygenase 1 and 3 in the liver [37]. Elevated circulating levels of TMAO increase the risk of cardiovascular diseases via promoting atherosclerotic lesion development; however, there is still controversy over it [58]. Some amino acids metabolites can serve as neurotransmitters. The gut microbiota can synthesize phenylalanine and tyrosine derivative dopamine via decarboxylation of *L*-DOPA by tyrosine decarboxylase [59]. Dopamine can be converted into norepinephrine and epinephrine via hydroxylation and methylation, respectively. Other neurotransmitters produced by gut microbiota include serotonin (5-hydroxytryptamine (5-HT)), norepinephrine, and *γ*-aminobutyric acid (GABA) [27].

In addition to metabolites mentioned above, the gut microbiota is capable of producing a series of other metabolites that are important for physiological functions of hosts. The gut microbiota can produce gases including hydrogen (H_2_), methane (CH_4_), carbon dioxide (CO_2_), hydrogen sulfide (H_2_S), nitric oxide (NO) that can modulate the physiology of hosts [28]. Lipids play important roles in the health of hosts, and the gut microbiota can produce lipopolysaccharides (LPS), conjugated fatty acids and other lipids to modulate the functions of hosts [7]. Assessment of the genomes of human gut bacteria for biosynthesis pathways of B-vitamins showed that 40-65% of the bacteria have the capability to synthesize vitamins, and the gut microbiota is an important producer of B-vitamins [60]. It should be noted that, although the importance of gut microbiota metabolites has been well recognized, the number of them is so huge that many of them remain uncharacterized. Therefore, mining of metabolites with special functions from the gut microbiota have become an important territory [61], and many methods for discovery of these metabolites have been developed, such as functional metagenomics, sequence-based (meta)genomics, and metabolomics [6,62,63].

### 2.3. Ways gut microbiota metabolites act on targets

**Figure 1.**
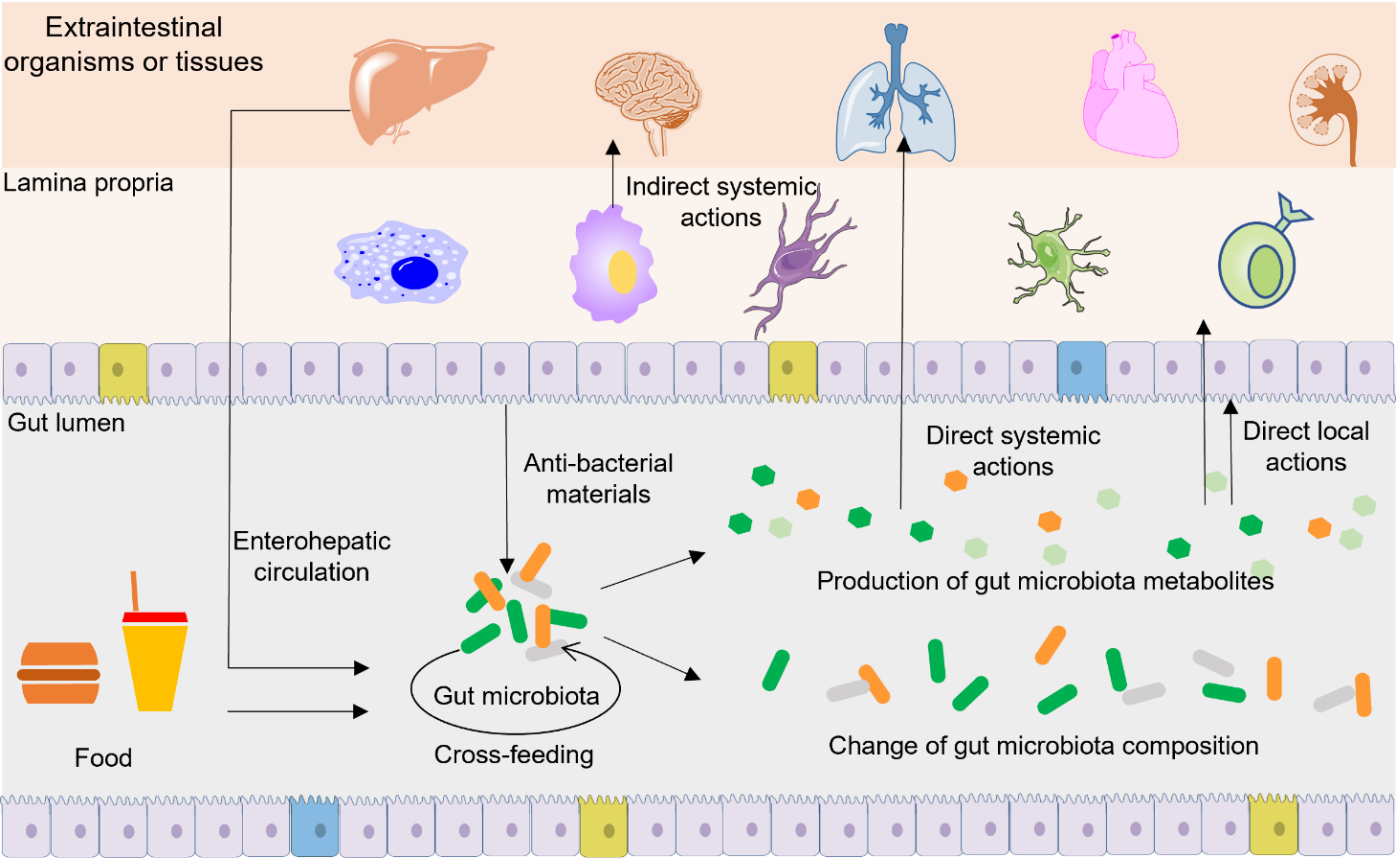
**Ways gut microbiota metabolites act on targets**. Within gut lumen, gut microbiota metabolites serve as the nutrients for some bacteria and change the composition of gut microbiota. Locally, gut microbiota metabolites can act on intestinal epithelium and immune cells in the lamina propria, and the local effects can further induce downstream systemic functions. Systemically, gut microbiota metabolites can be absorbed and transported to remote organs and tissues to exert diverse functions. Some gut microbiota metabolites can indirectly regulate the composition and function of gut microbiota via inducing hosts to synthesize and release anti-bacterial materials into gut lumen. Some gut microbiota metabolites may undergo enterohepatic circulation.

Gut microbiota metabolites act in multiple ways to directly or indirectly influence the functions of hosts (Fig. 1). The composition of gut microbiota is a key determinant in human health and disease, and the metabolism of gut microbiota is closely linked to the composition of gut microbiota. The gut microbiota metabolites can target on gut bacteria or hosts to regulate the composition and function of gut microbiota directly or indirectly. For example, gut microbiota metabolites SCFAs can serve as energy source for gut microbiota, and a high concentration of SCFAs can inhibit the growth of some gut bacteria [64]. In addition, SCFAs can modulate the production of secretory immunoglobulin A (sIgA), a non-inflammatory antibody synthesized by hosts, to prevent the invasive of pathogens [65]. Correspondingly, gut microbiota metabolites can modulate the composition of gut microbiota to indirectly influence the functions of hosts. Gut microbiota metabolites can also act on targets of host that are near or distant from gastrointestinal tract to directly modulate the functions of hosts. For example, upon their release into the gastrointestinal tract, SCFAs are directly sensed by the intestinal epithelial cells to influence the function of gut barrier [66]. SCFAs can also be delivered to tissues and organs that are remote from the gut, and then directly sensed by target tissues and organs to trigger extensive physiological changes of hosts [67]. In addition to SCFAs, other gut microbiota metabolites can also act directly or indirectly on targets that are within, near, or distant from gastrointestinal tract. We will discuss these specific mechanisms in the following section.

Bacterial cross-feeding refers to the process that one bacteria uptake or exchange the bacterial products with another [68]. Lactate, an end product of *Bifidobacterium*, is the typical bacterial product of cross-feeding. *Eubacterium hallii* cannot grow in pure starch condition. When *E. hallii* and *Bifidobacterium adolescentis* are co-cultured, significant reduction of lactate can be observed whereas butyrate shows an increase of concentration [69]. Thus, *E. hallii* can synthesis butyric acid using the lactose released by *B. adolescentis*. Cross-feeding provides significant advantages for bacteria in poor nutrient conditions and modulates antibiotic tolerance in bacterial communities, and thus influences the composition and function of gut microbiota [68,70,71].

## 3. Functions of gut microbiota metabolites

### 3.1. Regulation of the composition and function of gut microbiota

In gastrointestinal tract, a normal gut microbiota composition act as a barrier to defend the invasion of pathogens by stimulating hosts to secrete compounds with antimicrobial effects, competitive consumption of nutrients, and occupation of attachment sites [72]. On the contrary, dysbiosis, an abnormal change of gut microbiota composition, is associated with the development of a large number of diseases [73]. In addition to aforementioned SCFAs, many gut microbiota metabolites can directly modulate the composition and function of gut microbiota. For example, indole and indole derivatives can function as interspecies signaling molecules with the capability to regulate virulence, antibiotic resistance, biofilm formation, motility, and sporulation of gut bacteria [74]. Due to the detergent properties, bile acids can reshape the composition of gut microbiota by inhibiting the growth of gut bacteria via destroying the structure of bacterial membranes [22]. Among all the gut microbiota metabolites, of particular interest are antibiotics such as ribosomally synthesized, posttranslationally modified peptides such as lantibiotics, bacteriocins, microcins [46,48]. These metabolites are often toxic for a limited spectrum of bacterial species and are therefore hoped to be developed as new generation of antibiotics in the event of global abuse of antibiotics [75]. Besides these direct roles, gut microbiota metabolites can modulate the immune systems of hosts to indirectly affect the composition and function of gut microbiota (Fig. 1). For example, sIgA is a non-inflammatory antibody specialized in mucosal protection via maintenance of non-invasive commensal bacteria and neutralization of invasive pathogens [76]. SCFAs can improve the production of cecal sIgA and thus modulate the composition of gut microbiota [77]. Similarly, bile acids can act on FXR, thereby inducing the transcription of antimicrobial agents such as inducible nitric oxide synthase and interleukin (IL)-18 to regulate the composition of gut microbiota [78].

### 3.2. Serving as nutrition and influencing nutrition absorption

Gut microbiota such as *Bifidobacteria* can *de novo* synthesize a series of vitamins especially vitamin K and B group vitamins [79]. These vitamins can not only maintain the basic functions of bacterial metabolism, but also act as complementary endogenous sources of vitamins to maintain the metabolic and physiological functions of mammals. SCFAs can be directly degraded to provide energy or be used as resources for gluconeogenesis and lipid biosynthesis [20]. Gut microbiota amino acid metabolites including tryptophan, leucine, valine, and isoleucine are essential amino acids required for protein synthesis and are precursor of metabolites that can significantly affect mammalian physiology [80]. Minerals have many important functions in human, such as being a part and parcel of enzymes, hemoglobin, and bones. The factors influencing the bioavailability of minerals include synergism and antagonism between different minerals, presence of complexing or chelating compounds in the food, acidity of the colon, processing methods of food, health state of the organisms, environmental pollution, and gut microbiota [81]. Gut microbiota metabolites especially SCFAs can affect the bioavailability of minerals by lowering the acidity of intestinal luminal contents and changing the intestinal tissue morphology and transport proteins [82]. As the amphipathic and water-soluble molecules, bile acids emulsify dietary lipids and fat-soluble vitamins to facilitate the absorption of these nutrients after their release into the duodenum from the gall bladder [8].

### 3.3. Modulation of host metabolism

**Figure 2.**
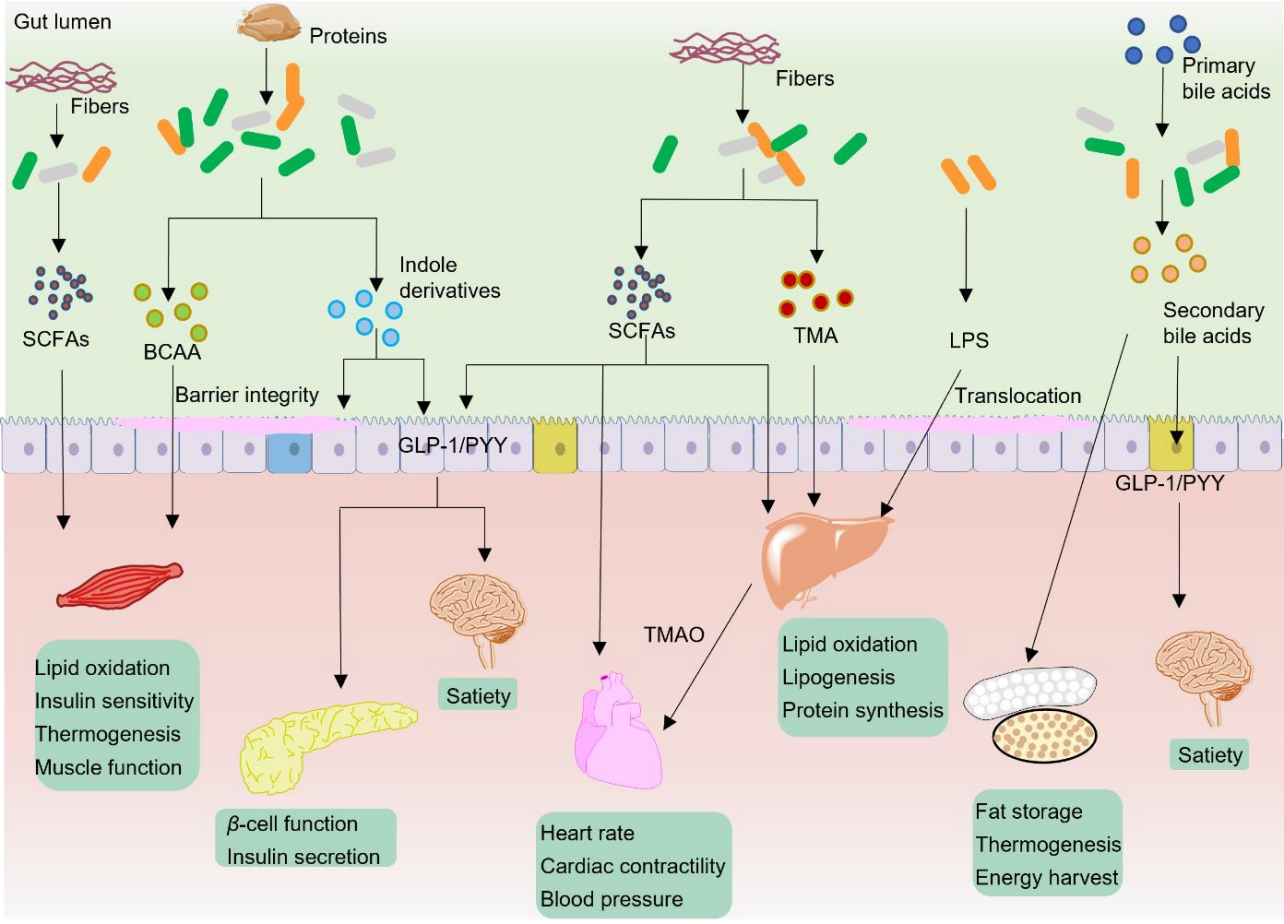
**Typical gut microbiota metabolites in modulation of host metabolism**. The major ways of this modulation include regulation of nutrition metabolism (lipids, proteins, glucose), non-shivering thermogenesis (browning of WAT and BAT), satiety (by secretion of hormone GLP-1 and PYY), motility function of organs (muscle and heart), insulin synthesis and secretion, and insulin sensitivity. By these ways, gut microbiota metabolites can maintain the homeostasis of energy.

Energy homeostasis, or the balance of energy storage and release, is vital for the survival and health of organisms. In adult mammals, long-term positive energy balance can result in obesity and increase the risks for various diseases such as type 2 diabetes, hypercholesterolemia, asthma, arthritis, liver diseases, and colon cancer [83,84]. On the contrary, long-term negative energy balance can lead to a number of diseases or physical disorders, including loss of bone mass, decline of thyroid hormones, reduction of physical performance and fertility [85,86]. Nutrients such as lipids and glucose have critical roles in energy metabolism and disease development. Over the past two decades, numerous studies have demonstrated that gut microbiota and gut microbiota metabolites play crucial roles in host metabolism via regulation of non-shivering thermogenesis, nutrition metabolism, satiety, motility function of organs, insulin synthesis and secretion, and insulin sensitivity (Fig. 2).

Adipose tissues play important roles in energy homeostasis. In general, adipose tissues are made up of three different tissues, i.e., brown adipose tissue (BAT), white adipose tissue (WAT) and beige adipose tissue. BAT and beige adipose tissue are major contributors to non-shivering thermogenesis in mammals while WAT is the key adipose tissue compartment to energy storage [87]. SCFAs, especially acetate, can inhibit lipolysis, increase adipogenesis, and thus improve the capacity of lipid storage of adipose tissues [88]. In cold environment, butyrate can partially rescue the impaired thermogenesis induced by antibiotics via improving the thermogenesis of BAT and browning of WAT [89]. The functions of insulin and *β*-cell are important for maintaining the blood glucose and the development of type II diabetes mellitus (T2DM). SCFAs has beneficial effects on *β*-cell function and insulin secretion via GPR43 [90,91]. The skeletal muscle is the largest organ in the human body and occupies a proportion of approximate 40% body mass, 30% resting energy expenditure, and 80% insulin-related glucose uptake [92,93]. SCFAs can influence the lipid, carbohydrate, and protein metabolism of skeletal muscle via acting on GPR41, GPR43, HDACs [94]. In the heart, SCFAs can act as an energy resource and acutely reduce the heart rate, cardiac contractility, and blood pressure [95,96]. Liver is an important metabolic organ for lipid and glucose metabolism. SCFAs in the liver can directly act as sources of energy. Propionate can be used for synthesis of glucose in liver while acetate can be used as substrates to synthesize cholesterol and long-chain fatty acids [97]. SCFAs can increase energy expenditure and decrease hepatic steatosis via inducing a switch from hepatic lipogenesis to hepatic beta-oxidation thereby protect against high-fat diet-induced obesity [98].

In addition to SCFAs, other gut microbiota metabolites also play important roles in regulation of energy metabolism. Activation of bile acid targets FXR and TGR5 can increase liver glycogen synthesis and insulin sensitivity, promote pancreas insulin secretion, facilitate energy metabolism in the liver, brown adipose tissue and muscles [99-101]. Like SCFAs, bile acids can act on enteroendocrine cells and facilitate the release of gut hormones such as glucagon-like peptide-1 (GLP-1) and peptide YY (PYY), which are involved in regulation of appetite and gut motility [8,102]. In the liver, bile acids can regulate triglyceride metabolism, especially the production of very low-density lipoprotein and lipogenesis, whose dysbolism further promote obesity, T2D as well as atherosclerosis and other cardiovascular diseases [103]. The branched-chain amino acids (BCAAs) are essential amino acids synthesized by gut microbiota. In the 1960s, researchers have already found out that BCAAs have direct roles on stimulation of insulin secretion and elevated levels of BCAAs are correlated with obesity and serum insulin [104,105]. One study showed that feeding mice a diet with specifically reduced BCAAs can improve glucose tolerance and body composition [106]. The mechanism of BCAAs in increasing energy expenditure is associated with chronic phosphorylation of mammalian target of rapamycin, c-Jun NH2-terminal kinase, and insulin receptor substrate 1 at 307 residue and by accumulating multiple acylcarnitines in the muscles [107]. Many studies have demonstrated the important roles of BCAAs in regulating protein synthesis, glucose and lipid metabolism, insulin resistance, hepatocyte proliferation, and thermogenesis of BAT [108]. Other gut microbiota metabolites such as TMAO, LPS, tryptophan and indole-derivative metabolites are closely linked with energy and nutrients metabolism [99]. Disturbed production of these gut microbiota metabolites is linked to a series of diseases such as obesity, T2DM, dyslipidemia, nonalcoholic fatty liver disease, atherosclerosis, and heart failure [109-112].

### 3.4. Influencing the intestinal barrier and gut motility

With a total surface of 200 m^2^, the gastrointestinal tract is the most exposed body system to the outside world [113]. Intestinal barrier is a layer of microbial, chemical, physical and immune barrier between the gut lumen and mucosal tissues with the critical functions of nutrient absorption and immune modulation. Specifically, the structure of intestinal barrier includes the outer microbial layer with gut commensals, mucus layer with the antimicrobial peptides and secretory immunoglobulin A (sIgA), the central single cell layer with epithelial cells (absorptive enterocytes, Goblet cells, enteroendocrine cells and Paneth cells), and the inner lamina propria layer where innate and adaptive immune cells dwell, such as T cells, B cells, dendritic cells, macrophages, and IgA producing plasma cells [5,114,115]. The permeability of the epithelium is determined by the tight junctions that reside near the apical surface of adjacent epithelial cells [116]. Increased intestinal epithelial permeability, also called “leaky gut”, facilitates the translocation of harmful substances such as LPS and pathogens to the inner layer of intestinal barrier and bloodstream [117]. Translocation of these materials can further affect the immune and metabolomic status of hosts and is associated with a wide range of diseases, including celiac disease, colorectal cancer, irritable bowel syndrome, inflammatory bowel disease and other extraintestinal diseases such as chronic liver diseases, T1DM, obesity and food allergy [5,118,119].

**Figure 3.**
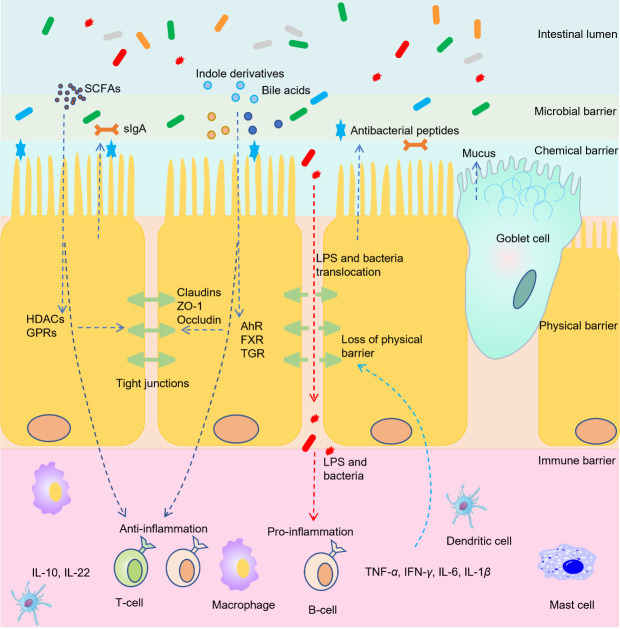
**Gut microbiota metabolites modulation of intestinal barrier**. Intestinal barrier consists of microbial barrier, chemical barrier, physical barrier, and immune barrier. SCFAs can enhance the chemical barrier by stimulating the secretion of antimicrobial peptides, sIgA and mucins to prevent harmful bacteria. SCFAs, bile acids, and indole derivatives can enhance physical barrier via increasing tight junction proteins such as cludins, occluden-1, and occludin. The epithelial cross of SCFAs and indole derivatives can act on immune cells and lead to release of anti-inflammatory cytokines such as IL-10 and IL-22. During chronic diseases, disturbance of tights junctions can lead to destruction of physical barrier, and further lead to translocation of LPS and bacteria. This translocation triggers the activation of immune cells and lead to production of pro-inflammatory cytokines. The release of pro-inflammatory cytokines can act on local epithelial cells to worsen physical barrier or can act on extraintestinal organs to trigger other diseases.

The function of intestinal barrier is largely affected by gut microbiota metabolites (Fig. 3). SCFAs especially butyrate can orchestrate the genes encoding tight-junction proteins and regulate the redistribution of occludin to prevent abnormal intestinal permeability [120]. SCFAs can also regulate intestinal barrier by stimulating the hosts to secrete antimicrobial peptides, sIgA and mucins to prevent the adherence or invasion of harmful bacteria [121,122]. The immune cells in intestinal barrier secrete inflammatory cytokines such as IL-10, IL-1 *β*, IL-6, IL-22, tumor necrosis factor α (TNF- *α*) to maintain the normal functions of immune system. On the contrary, excessive production of these cytokines can lead to systemic inflammation and diseases. SCFAs exert anti-inflammatory effects by regulating cytokine production and immune cell functions [123]. Indole and indole derivatives can act as ligands of epithelial nuclear receptors such as AhR, PXR and retinoid-related orphan receptor gamma-t to affect the functions of intestinal barrier [33]. For example, indole can enhance the trans-epithelial resistance of epithelial cells, induce the expression of intestinal tight junction proteins, decrease the inflammatory cytokines IL-1 *α*, IL-1 *β*, TNF- *α* and IL-6 [33,36]. Activation of bile acid target TGR5 by BAR501 shifted the macrophage phenotypes from M1 (pro-inflammatory) to M2 (tissue-protective), decreased the expression of proinflammatory cytokines including TNF- *α*, IFN- *γ*, IL-1 *β*, IL-6, and enhanced the expression of anti-inflammatory cytokines including transforming growth factor *β* and IL-10 [124].

The proper function of intestinal motility is important for digestion, absorption, and secretion of nutrients and wastes whereas dysmotility of intestine can lead to infections, malabsorption of nutrients, diarrhea, and constipation [125,126]. Slow colonic transit is associated with increased bacterial production of methane, protein catabolism, carbohydrate deprivation, and an increase of detrimental metabolites such as ammonia and aromatic derivatives of amino acids [127]. On the contrary, fast transit is associated with increased gut health and reduced low-grade intestinal inflammation [128]. Gut microbiota metabolites such as SCFAs, LPS, secondary bile acids and methane can interact with enteric nerves and smooth muscles via neural and humoral pathways such as GLP-1, PYY, motilin, and serotonin to modulate gastric emptying and colonic motility [129,130].

### 3.5. Impacting the systemic immune response

In addition to modulate the local intestinal immunity, gut microbiota can also influence both adaptive and innate immune responses in multiple extraintestinal organs via acting on a plethora of immune cells such as B cells, T cells, and macrophages. In spleen B cells, SCFAs support antibody production via increasing acetyl-CoA and regulating metabolic sensors [77]. In addition, mice with low SCFA production showed defects in homeostatic and pathogen-specific antibody responses, leading to bigger susceptibility to pathogens whereas SCFA intake can restore this immune deficiency [77]. In the lung, gut microbiota metabolites predominately SCFAs are responsible for the protection against allergy and asthma. Antibiotics treatment leads to elevated allergic lung inflammation whereas administration of SCFAs reduces this inflammation by manipulation of T helper type 2 cells and decreasing the circulating immunoglobulin E [67,131]. In pregnant mice, administration of acetate in drinking water protected offspring from asthma during adulthood, and the mechanism is associated with regulating mucosal immune responses via stimulation of GPCRs such as GPR109A and GPR41 [132], and inhibition of HDACs to enhance the number and function of Treg cells [133]. Butyrate protected the lungs of influenza-infected mice via regulating the metabolism of influenza-specific CD8+ T cells and shaping Ly6c- patrolling monocyte hematopoiesis [134].

Autoimmune cholestatic liver diseases, including primary sclerosing cholangitis and primary biliary cholangitis, are a type of diseases that show impairment of bile flow and excessive accumulation of toxic bile acids [135]. In the liver, high concentrations of bile acids contribute to autoimmune liver injury and can result in activation of pro-inflammatory programs and production of NF- *κ*B dependent mediators [136-138]. In addition, bile acids can cause direct injury on biliary epithelial cells and lead to production of IL-6 and IL-1 *β*, which will further result in augmentative inflammation [139]. Non-alcoholic fatty liver disease is a metabolic liver disease characterized by hepatic steatosis and can progress to non-alcoholic steatohepatitis that shows obvious hepatic inflammatory damage [140]. SCFAs such as acetate can alleviate hepatic inflammation by suppression of the macrophage proinflammatory activation [141].

Increased level of TMAO is positively correlated with atherosclerosis and cardiovascular diseases such as myocardial infarction, hypertension, and myocardial fibrosis [142]. TMAO can impair endothelial self-repair capacity and enhancing monocyte adhesion via activating NF- *к*B, protein kinase C and nucleotide-binding oligomerization domain-like receptor family pyrin domain-containing 3 (NLRP3) inflammasome [143]. In mice, voluntary exercise inhibited cardiac dysfunction in western diet-induced obesity via preventing myocardial inflammation and fibrosis [144]. However, TMAO supplementation abolished this cardioprotective effects of voluntary exercise via inducing myocardial inflammation by increasing TNF- *α* and IL-10 [144]. In mouse models of doxorubicin-induced cardiac fibrosis, TMAO aggravated cardiac fibrosis by activating NLRP3 inflammasome. On the contrary, silencing of NLRP3 protected the mice from cardiac fibrosis as evidence by amelioration of cellular proliferation, migration and collagen deposition [145].

Osteoporosis is a skeletal disease that is characterized by low bone mineral density and microstructural destruction of bone tissues. In the bone, gut microbiota metabolites such as LPS, SCFAs, and bile acids can influence the immune function to modulate the bone metabolism [146]. For example, increased permeability of intestinal barrier causes more LPS into the circulation system and reduction of bone mineral density [147]. The mechanisms are associated with activation of TLR2 and TLR4 in mesenchymal stromal cells to inhibit osteoblastic differentiation, activation of NF- *κ*B and MAPK signaling pathways to increase the differentiation of macrophages into osteoclasts [148]. Plasma metabolome study demonstrated the existence of gut microbiota-derived uraemic toxins, including LPS, tryptophan derivative indoxyl sulfate, tyrosine or phenylalanine derivatives *p*-cresol sulfate and phenylacetylglutamine [149]. In the kidney, these metabolites can accumulate and contribute to pathogenesis and progression of chronic kidney disease (CKD) via triggering renal damage, inflammation and fibrosis [150].

In addition to the effects mentioned above, gut microbiota metabolites can also affect other organs or tissues to influence the development of diseases such as systemic lupus erythematosus, rheumatoid arthritis, human immunodeficiency virus, and inflammatory skin diseases [151-153]. For example, rheumatoid arthritis is a disease caused by the malfunction of white blood cells and it affects approximately 1% of the population worldwide [154]. Butyrate supplementation ameliorates arthritis in a regulatory B cells-dependent way via elevating the content of the serotonin derivative 5-hydroxyindole-3-acetic acid, a compound that can further activate AhR, a transcriptional marker for regulatory B cell function [154]. Gut microbiota metabolites can also influence the immunity of nervous system, which we will discuss in the next section.

### 3.6. Influencing the nervous system

**Figure 4.**
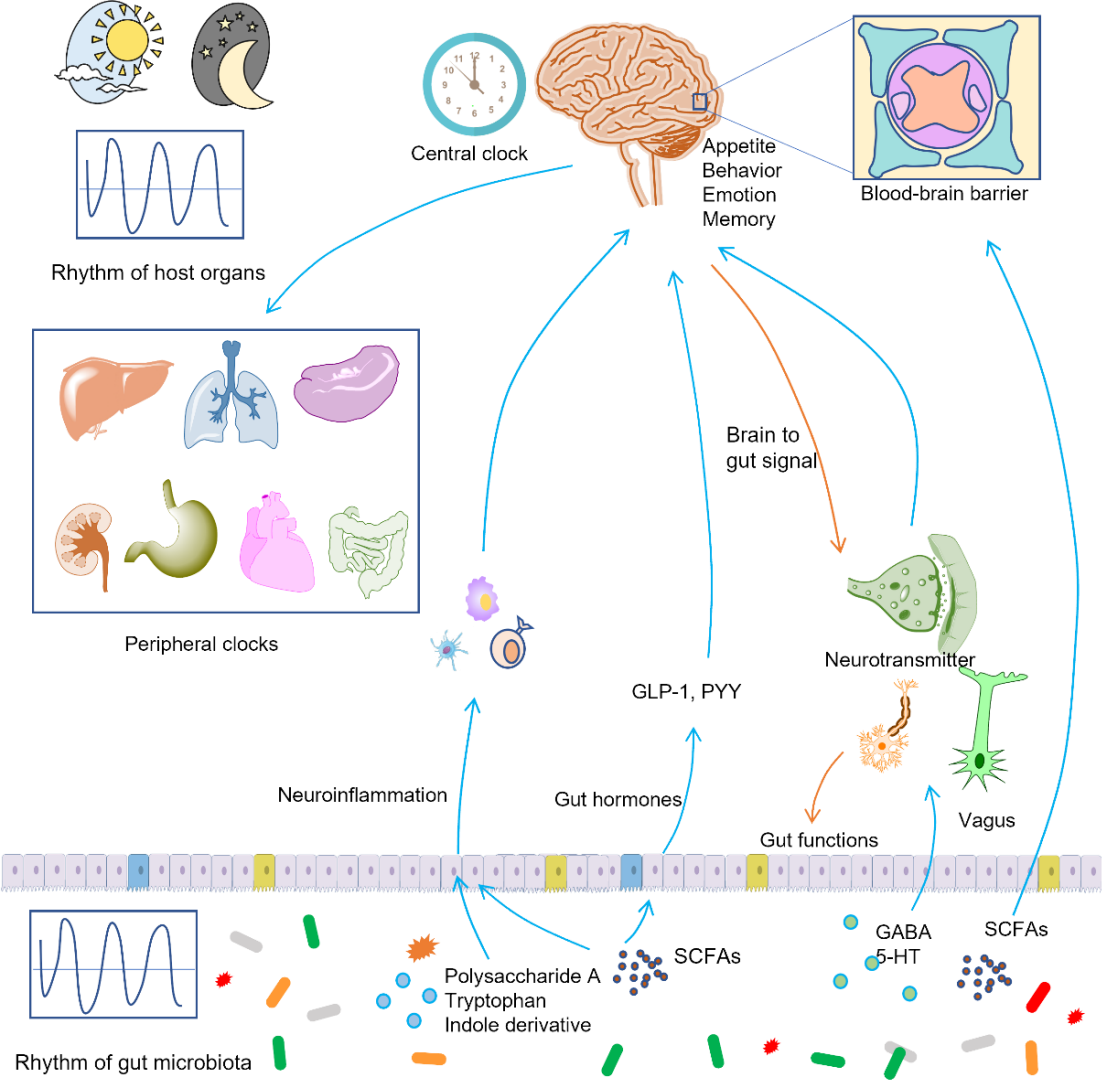
**Gut microbiota metabolites modulate the nervous system and circadian rhythm**. Gut microbiota metabolites can modulate the cerebral inflammation, the production of gut hormones, the transmission of nervous impulse, the functions of blood-brain barrier to regulate the functions of brains such as the emotion and appetite. On the contrary, the brain can modulate the functions of gut such as the gut motility and secretion of digestive juice. The production of gut microbiota metabolites shows day-night rhythm, and this rhythm can be transported to the central clocks and peripheral clocks to modulate the systemic functions of hosts. Similarly, the central clock can transfer the light and dark cues to peripheral organs and gut microbiota, and thus synchronize the functions of gut microbiota and host clocks.

It has long been established that central nervous system (CNS) can modulate the functions of gut such as gut motility, digestive juice secretion, immune function, blood flow, and nociception [155,156]. In addition, the signaling from brain to gut can further affect the composition and function of gut microbiota via intestinal immune system [157]. On the contrary, a growing body of evidence emerged in recent years suggests that the gut microbiota metabolites exert significant influences on brain (Fig. 4), and thus affect the development of neurological and psychiatric disorders such as multiple sclerosis, major depressive disorder, anxiety disorder, Parkinson’s disease, Alzheimer’s disease, and autism [155]. For example, bacteria-derived LPS can act as the mediator to trigger inflammatory bowel disease-related psychosocial disturbances [158].

The blood-brain barrier (BBB) is part of a neurovascular unit that comprise brain microvascular endothelial cells, astrocytes, neurons, pericytes, extracellular matrix, and microglia. BBB plays essential roles in preventing the entrance of therapeutic drugs to brain and protecting the brain from injuries and diseases by tightly segregating the brain from the circulating blood. Infections, autoimmune diseases, and brain injury can change the integrity of BBB, rendering the increased accessibility of the brain to harmful products in the circulatory system [159]. SCFAs play important roles in maintaining the integrity of BBB as evidenced by the fact that intravenous or intraperitoneal administration of sodium butyrate can inhibit BBB breakdown following the traumatic brain injury [160]. SCFAs can cross BBB via transporter H^+^-dependent or sodium-dependent monocarboxylate transporters that are abundantly expressed in endothelial cells [50]. In the brain, SCFAs can directly regulate learning, memory, behavior, and disease progress [161]. In addition to SCFAs, many other gut microbiota metabolites such as BCAAs, GABA, and indole derivatives can cross BBB to exert the extensive effects on brain [162].

The CNS contains a variety of innate and adaptive immune cells including microglia, astrocytes, perivascular macrophages, CD4+ T and CD8+ T cells, and mast cells that can affect cerebral inflammation [155]. Usually, physiological production of cytokines by activation of CNS immune cells causes minimal impact on the CNS. On the contrary, chronic systemic inflammation can lead to significant behavioral alterations and cognitive dysfunctions [163,164]. Administration of mice with SCFAs reduced experimental autoimmune encephalomyelitis and axonal damage through increasing Treg differentiation by suppression of the c-Jun NH2-terminal kinase 1 and p38 pathway [165]. Similarly, treatment with polysaccharide A, a gut bacterial product, protected the wild-type mice against CNS demyelination and inflammation by a Toll-like receptor (TLR)-2-dependent pathway [166]. Astrocytes are one type of glial cells that present abundantly in the brain and can modulate neural inflammatory responses by cytokine production and antigen presentation [167]. The gut microbiota metabolites tryptophan and indole derivative can activate AhR to modulate astrocyte activity, and thus influence the multiple sclerosis in animals [168].

Feelings of hunger and satiety are important motivations for feeding behavior. Gut microbiota metabolites can influence the appetite, and thus maintain the metabolic health of hosts or cause a number of metabolic disorders. In the colon, gut microbiota metabolites can promote enteroendocrine cells to produce anorexigenic hormones (PYY, GLP-1, and cholecystokinin), neurotransmitter 5-HT, and peripheral hormones (leptin, ghrelin, and insulin) [169]. For example, SCFAs can binding to GRP43 and thus lead to the release of GLP-1, PYY, insulin, and leptin [170,171]. The anorexigenic hormones including GLP-1 and PYY can cross the BBB and activate proopiomelanocortin in the brain [172,173]. In addition, some gut microbiota metabolites such as GABA can act as neurotransmitter to regulate appetite. This is supported by the fact that disruption of GABA signaling pathways suppressed postweaning feeding, blunted neuropeptide Y-triggered hyperphagia, and hunger-associated appetite [174,175]. Furthermore, gut microbiota can synthesis protein sequences that are identical to peptides with appetite-regulating effects, such as caseinolytic protease, a mimic of alpha-melanocyte-stimulating hormone to induce anorexigenic effects [176].

### 3.7. Modulation of circadian rhythm

Human is evolved to adapt to a circadian rhythm of ~24 h that is in concert with the light/dark cycle on the earth. Circadian rhythms affect the microscopic molecular oscillations in genes, proteins, and metabolites and macroscopic aspects of biology and physiology such as behavior, sleep-wake cycles, gastrointestinal digestion, absorption, motility, and hormone secretion [177,178]. The ‘central’ circadian clock is located in the suprachiasmatic nucleus of hypothalamus. It receives environmental light and dark cues and synchronizes this information to ‘peripheral’ clocks in peripheral tissues to keep the body functioning in a same rhythm [179]. Circadian rhythms can be disrupted by lifestyle factors such as shift work, jet lag, sleep deprivation, artificial light at night, food type, and timing of food consumption [180]. Disruption of circadian rhythms is implicated in a number of diseases such as neurodegenerative diseases, cardiovascular diseases, gastrointestinal diseases, metabolic diseases, sleep and psychiatric disorders, and cancer [181-185].

Recent studies showed that gut microbiota can regulate or be regulated by the central and peripheral circadian clocks (Fig. 4). Like mammals, gut microbiota exhibits circadian rhythm and shows compositional and functional oscillation, and this oscillation further programs host transcriptome oscillations that eventually affect hepatic drug detoxification and drug hepatotoxicity [186]. The mechanisms that gut microbiota can affect host circadian rhythms include contact-dependent and contact-independent mechanisms [187]. The contact-dependent mechanisms need a direct contact between gut bacteria and gastrointestinal cells and the following activation of pattern recognition receptors such as NOD-like receptors and TLRs [187]. The contact-independent mechanisms mainly require small molecular gut microbiota metabolites such as bile acids and SCFAs to act as mediators [179]. For example, the levels of butyrate and propionate show obvious diurnal oscillation, and this oscillation is lost under high-fat feeding [188]. Treatment of murine and hepatic-derived organoids with individual SCFAs significantly changed the oscillations of expression of major hepatic circadian genes including *Bmal1* and *Per2*[188]. On the contrary, deletion of *Bmal1*abolished the rhythmicity of fecal SCFA levels, supporting that circadian rhythm of hosts can regulate the rhythmicity of gut microbiota [189].

The effects of gut microbiota metabolites on circadian rhythm are vast and are necessarily interconnected with other functions of gut microbiota metabolites such as modulation of energy metabolism and immune response. These interactions between different functions via the nexus of gut microbiota metabolites are important for understanding the functions of gut microbiota metabolites holistically. For example, gut microbiota programs diurnal oscillations of a SCFAs receptor HDAC3 and thus produce synchronized diurnal oscillations in histone acetylation [190]. The oscillations of histone acetylation further regulate metabolic gene expression and nutrient uptake in a diurnally oscillatory manner. In addition, the oscillations of HDAC3 induced rhythmic transcription of the lipid transporter gene *Cd36*and high-fat-diet-induced obesity [190]. The study demonstrated the tight relationships of different functions of gut microbiota metabolites.

### 3.8. Affecting drug efficacy and toxicity

Gut microbiota plays important roles in modification of the toxicity and efficacy of drugs and herbal compounds such as metformin, berberine, aconitine [4]. Gut microbiota metabolites can compete with drug-metabolizing enzymes, affect the expression of drug transporters and hepatic drug-metabolizing enzymes to modulate the efficacy and toxicity of drugs. *p*-Cresol is a microbial product of tyrosine and phenylalanine by organisms that belong to *Firmicutes, Bacteroidetes*, *Actinobacteria* and *Fusobacteria* phyla [191,192]. Acetaminophen is a widely used non-steroidal anti-inflammatory drug to treat pain and fever. Overdose of acetaminophen can cause severe and sometimes fatal hepatotoxicity in clinic [193]. In the liver, the toxicity of acetaminophen can be reduced by transforming it into inactive acetaminophen sulfate, whereas a small portion of it can be transformed into toxic compound *N*-acetyl- *p*-benzoquinone imine by cytochrome P450 (CYP) 2E1 and CYP3A4 [194,195]. *p*-cresol and acetaminophen can compete for human cytosolic sulfotransferase 1A1, an enzyme responsible for transforming *p*-cresol to inactive compounds. This competition hampers the capability of liver to reduce the toxicity of acetaminophen [196]. Drug transporters are membrane proteins that transport a wide range of materials into and out of cells and play significant functions in the drug absorption, distribution, and excretion. They are expressed in many tissues such as the intestine, liver, kidney, and brain [197]. Butyrate can downregulate the expression and function of drug transporter P-glycoprotein via inhibiting HDAC/NF- *κ*B pathways [198]. Hepatic metabolism of xenobiotics is mainly regulated by nuclear receptors such as constitutive androstane receptor and PXR [199]. Modulation of AhR by tryptophan and indole derivatives many further modulate the transcription of hepatic and duodenal CYP450 1a genes [200]. The modulation of metabolic enzymes and drug transporters may further impact the metabolism of drugs. However, more research is needed to support this hypothesis.

## 4. Considerations and perspectives

In addition to the metabolites we have mentioned, the gut microbiota has the potential to synthesize a large number of other structurally distinct metabolites. However, the structures and functions of these microbial metabolites, for the most part, remain unknown. For this reason, it is needed to mine these metabolites systemically. Currently, a variety of methods such as culture-based, genome/metagenomics-based, and metabolomics-based methods have been developed to mine the metabolites with potent bioactivities, and these methods have found plenty exciting achievements [61]. These metabolites discovered are hoped to exhibit super pharmacological effects on hosts and can be developed as novel antibiotics, immunoregulators, anti-inflammatory and anti-obesity drugs [201]. For example, traditional antibiotics can cause extensive damage to the gut microbiota composition and even lead to secondary infections [202]. On the contrary, the antimicrobial metabolites synthesized by gut microbiota such as bacteriocins can be developed as novel antibiotics because they can targeted inhibit or eliminate a certain strain [203]. In addition, the drugs mined from gut microbiota exhibit the advantage over other drugs since they can escape the chemical conversion of gut microbiota, a process that can strongly influence the drug efficacy and toxicity [204,205].

Precision medicine (personalized medicine, stratified medicine, person-centered medicine) is a field of medicine that aims to optimize the medical diagnosis and disease treatment by taking into account an individual's genes, microbiomes, environments, etc. Because of their significance in health and disease, gut microbiota has become an important consideration in the way toward precision medicine [206]. To achieve the goal of precision medicine, it is necessary to find suitable biomarkers for patient stratification and treatment decision at first. The biomarkers can be gut microbiota species, metabolites, microbial genes and enzymes that are responsible for the production of metabolites [4]. For example, SCFAs can be a potential biomarker for screening of patients with celiac disease, adenomatous polyposis, and colorectal cancer [207,208]. When the biomarkers are found, suitable methods are needed to modulate the gut microbiota. The conventional methods include fecal microbiota transplantation (FMT), diet, prebiotics, probiotics, synbiotics, and antibiotics. Recently, supplement of microbial enzyme inhibitors, gut microbiota metabolites, and probiotics have been developed as new methods for precision medicine. For example, butyrate-rich diets have recently been used for improving the redox status and fibrin lysis in Behçet’s Syndrome [209]. Targeted inhibition of microbial TMA production by 3,3-dimethyl-1-butanol non-lethally reduced the level of plasma TMAO in mice supplemented with a high-choline or L-carnitine diet [210]. More importantly, administration of 3,3-dimethyl-1-butanol can reduce the progression of atherosclerosis in Apoe^-/-^ mice, suggesting of the use of enzyme inhibitors in precision medicine [210].

Special attention should be paid to the biomarkers of precision medicine considering that the progresses of diseases are dynamic and so does the gut microbiota. During the disease development, the levels of biomarkers are dynamic and should be monitored repeatedly. Correspondingly, the methods for manipulation of gut microbiota, the dosage of drugs and enzyme inhibitors should be adjusted according to the levels of the biomarkers. More importantly, gut microbiota and hosts are highly interactive since some gut microbiota metabolites, such as TMA, can be further metabolized by hosts. Therefore, microbial-based biomarkers may lead to the failure of precision medicine. In order to develop more precise biomarkers, integrating information about hosts, gut microbiota and other external factors as integrative biomarkers is perhaps more important. For example, vedoNet, a comprehensive neural network algorithm incorporating clinical and microbiome-related data, is useful for predict patients’ response to inflammatory bowel disease treatment [211].

## 5. Conclusion

The gut microbiota and the host are involved in a complex crosstalk that is influenced by environmental conditions, and they interact with each other both locally and systemically. Owing to the diversity of gut microbiota and the gut microbial genes, the gut microbiota produces many structurally distinct metabolites. These metabolites shuttle between gut microorganisms and between gut microorganisms and hosts, thereby exert a wide range of bioactivities to modulate the functions of both gut microbiota and hosts. Because of the variability of gut microbiota, targeting of gut microbiota metabolites is a necessary procedure toward precision medicine. To achieve the aim of precision medicine, diagnostic microbial biomarkers or dynamic integrative biomarkers should be developed. Of note, although a large numbers of gut microbiota metabolites have been identified, systematic discovery of more microbial metabolites is needed, and these metabolites are hoped to be developed as new drugs for disease treatment.
